# Are Various Forms of Locomotion-Speed Diverse or Unique Performance Quality?

**DOI:** 10.2478/hukin-2013-0045

**Published:** 2013-10-08

**Authors:** Mile Cavar, Marin Corluka, Ivana Cerkez, Zoran Culjak, Damir Sekulic

**Affiliations:** 1 University of Mostar, Faculty of Natural Science, Mathematics and Education, Mostar, Bosnia and Herzegovina.; 2 University of Split, Faculty of Kinesiology, Split, Croatia.; 3 University of Split, Department of Health Care Studies, Split, Croatia.

**Keywords:** specific testing, factor analysis, bipedal locomotion, quadrupedal locomotion

## Abstract

The forward-sprint is considered to be, and is regularly performed as, a unique measure of “on-ground” linear-speed performance. Thus far, no investigation has simultaneously studied different forms of linear-speed or investigated whether different forms of linear-speed should be observed as unique performance quality. The purpose of this study was to determine (I) the achievements (i.e. execution time), and (II) the reliability and inter-relationships between various linear-speed performances. The participants were 42 male physical education students with substantial sport-specific backgrounds. We applied a total of six tests: three quadrupedal (supine backward, supine forward, and pronate backward locomotion) and three bipedal-performances (forward sprinting, backward sprinting, lateral shuffling). All of the tests showed appropriate reliability parameters (Cronbach Alpha ranged from 0.91 to 0.97; Inter-Item-R 0.78–0.92; Coefficient-of-Variation 1.3–9.1). The tests used in this study shared between 9% and 50% of the common variance. Our results suggest that different activities require activity-specific tests of linear-speed. This is particularly significant in those sports and activities in which quadrupedal locomotion patterns are highly important (wrestling, physically trained military services, law enforcement, fire and rescue, protective services).

## Introduction

In sport science and practice, linear speed is regularly defined as maximal forward running of a specific straight line distance (Salonikidis and Zafeiridis, 2008; Green et al., 2011). Linear speed is considered to be important not only in athletics (i.e., track and field) but also in many team and individual sports (Salonikidis and Zafeiridis, 2008; Green et al., 2011). Consequently, there is a particular interest in training and testing characteristic running speed. Based on previous studies (Salonikidis and Zafeiridis, 2008; Green et al., 2011; Alemdaroğlu, 2012; Rey et al., 2012), it can be concluded that characteristic demands of each sport, the athlete’s playing-position-specific duties, the sizes of the sport courts and fields, and task-specific linear-speed manifestations (i.e. acceleration or maximal speed) define the distance for linear-speed running tests.

The relationships between different sprint distances (from 10 to 40 m of straight-line-sprint from a static start) were rarely below r=0.78, and reached up to r=0.90 (Harris et al., 2008; Young et al., 2008; Salaj and Markovic, 2011). Hence, it seems that those tests assess similar qualities. In contrast, the popular perception of linear-speed as an exclusively forward-running manifestation does not take into account other sport and occupational activities that comprise some specific movement patterns that are frequently performed with maximal intensity and on a straight line course, but not throughout forward-running. For example, there are other bipedal sport-specific locomotions such as backward running in soccer, or the lateral shuffle in basketball and handball (Mohr et al., 2003; Dayakidis and Boudolos, 2006). The same should be observed for quadrupedal locomotions, such as crawling, creeping, etc., which are used in some tactical activities such as fire-fighting and the military services (Sheaff et al., 2010; Pandorf et al., 2003).

The literature shows that various bipedal and quadrupedal “non-forward-running” maximal locomotions are used by researchers as parts of agility tests (e.g., the backward running and lateral shuffle), obstacle courses (e.g., crawling) and coordination tests (e.g., the backward crawling) (Chaouachi et al., 2009; Sporis et al., 2010; Sekulic et al., 2012; Sheaff et al., 2010; Sekulic et al., 2006a). However, such types of locomotions are rarely observed as autonomous straight-line maximal locomotion. In contrast, although some non-forward running straight-line autonomous locomotions are used in kinematic-analyses and energy-expenditure-analyses (Abitbol, 1988; Flynn et al., 1994; Cavagna et al., 2011), they are regularly of submaximal intensity and are thus not discussed here. Consequently, to the best of our knowledge, the relationships between different types of locomotion forms have not been investigated.

From our point of view, it is crucial to find out whether those performances have specific qualities that should be tested and trained specifically, or whether we should observe a “universal” linear speed quality, regardless to different locomotion forms and movement specifics (forward, backward, lateral, bipedal, quadrupedal, etc.). This issue is particularly important in tactical activities, such as physically trained military, law enforcement, fire and rescue, protective services, and other emergency services for which those abilities are highly relevant (Faff and Korneta, 2000; Sekulic et al., 2006b).

Thus, the purpose of our study was to determine the interrelationships between various linear maximal short-distance performances, that consist of different movement patterns (running, lateral shuffle [running], backward running and three types of specific quadrupedal locomotion). We hypothesized that there are no strong relationships between very different forms of maximal locomotion irrespectively of their similar physiological background (i.e. ATP-CP energetic requirements).

## Material and Methods

### Participants

Forty-two healthy male physical education students (mean ± SD: age: 19.8 ± 1.3 years; body mass: 80.4 ± 9.6 kg; body height, 1.84 ± 0.07 m) participated in the present study. The participants had various sports backgrounds, which included team sports (soccer, handball, basketball), racquet sports, combat sports and dance sports. All of the subjects were involved in systematic sports training for at least five years. To avoid the possible negative effect of fatigue on the test procedure, the subjects were requested not to perform strenuous exercises 48 hours prior to testing and between the testing sessions.

### Measures

The variables in this study included six diverse linear short-distance performances of maximal intensity (three bipedal and three quadrupedal locomotions). Our objective was to obtain a similar physiological background for all of the tests. Therefore, all six tests were maximal with regard to their intensity and brevity (4–10 s), and the straight-line distances were 18 and 30 m depending on the movement efficacy of the locomotion form. Because of the higher movement-efficacy, the forward and backward running tests were performed over the longer distances in comparison to other tests. The subjects executed maximal performance without a signal to avoid the possible effects of reaction time of final achievement. The subjects performed three trials of each test (from a stationary start), with at least 3 min of rest between all trials and tests. The best performance was used for further analysis.

Bipedal testing: Bipedal tests consisted of 30 m forward sprint (S30), 30 m backward sprint (BS30), and lateral shuffle running test over an 18 m distance (LT). The (start) front foot in all three bipedal tests (S30, BS30, LT) was positioned 10 cm behind the first timing gate or the starting line. The height of electronic gates was set at 90 cm. The subject was positioned frontally to the photocell beam and in a sideways position for the lateral shuffle test (LT), with the leading foot arbitrarily selected and unchanged in all three trials. The subjects were reminded of the basic techniques for proper sprinting and lateral running. For example, during the LT, the subjects were not allowed to perform crossover steps, and during the S30 and BS30, they were especially advised not to decelerate before crossing the finish line.

**Figure 1:**
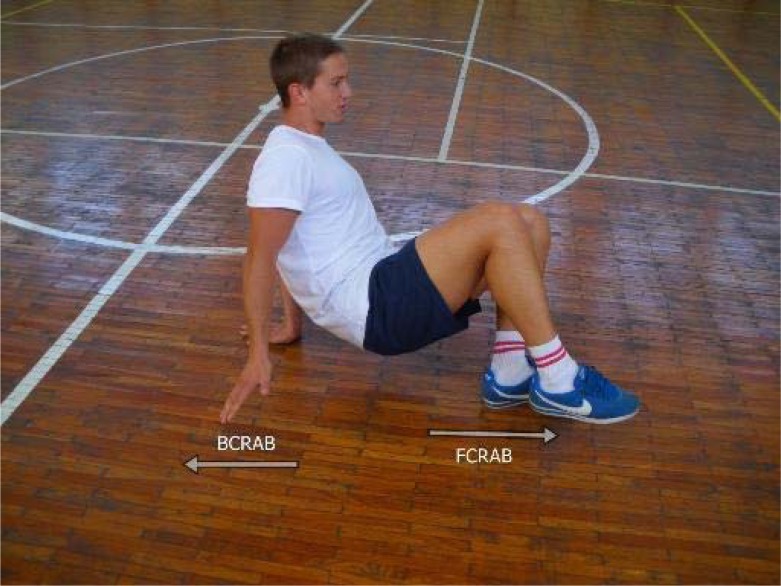
Performance of the forward crab walks (FCRAB) and reverse crab walks (BCRAB)

**Figure 2:**
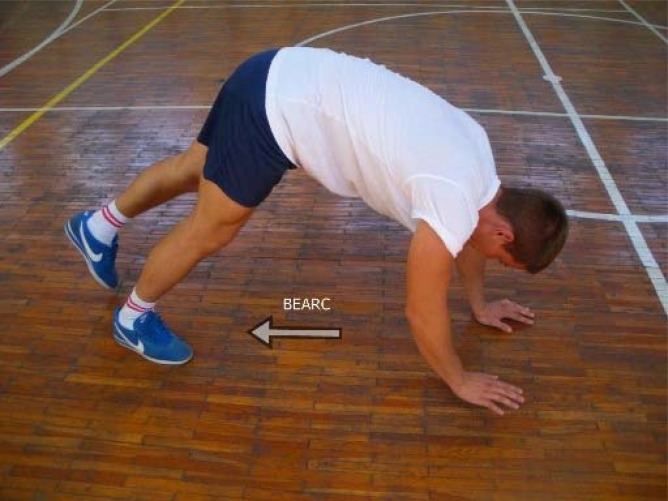
Performance of the reverse bear crawl (BEARC)

Quadrupedal testing: All of the quadrupedal performances (supine backward, supine forward and pronate backward locomotions) began from the stationary position, with both hands and feet touching the ground and were performed over the distance of 18 m. The electronic gates were positioned at 30 cm height, to match position of quadrupedal performances and lower center of gravity during such locomotion. The supine forward (FCRAB) and backward locomotion (BCRAB) tests consisted of locomotions that, in practice and in the literature, are known as the forward (FCRAB) and reverse crab walks (BCRAB) (Lauren and Clark, 2011). The tests were performed by simultaneously moving the same side foot and hand forward (Figure 1). The pronate quadrupedal locomotion, known as the Bear crawl (Grindstaff and Potach, 2006), in the backward direction (reverse bear crawl) was applied in the test BEARC (Figure 2). BEARC is performed by simultaneous movement of one hand and the opposite leg, which consequently results in a backward movement pattern. The applied quadrupedal tests were commenced with the feet (FCRAB and BEARC) or palms (BCRAB) positioned parallel to the line and 10 cm behind the first electronic gate.

### Procedures

Testing procedures were performed within university research laboratory by four experienced examiners using electronic timing gates (Brower Timing System, Salt Lake City, UT). All of the tests except S30 and BS30 were performed on a standard indoor wood floor. Due to limitations of the gymnasium length, S30 and BS30 were performed on an athletic track.

The subjects performed warm-ups prior to testing, including jogging, stability exercises and dynamic range of movement activities. The warm-up protocol also included a practice trial before each test performance. To minimize the effects of fatigue, the test procedure was conducted during a 2-day period (Monday and Thursday). On the first day of testing the subjects performed FCRAB, BCRAB and LT. On the second day S30, BS30 and BEARC were done.

Before participation, the experimental procedures were explained, and written consent was obtained from all of the subjects. The study was approved by the institutional ethics committee and met conditions of the Declaration of Helsinki.

### Statistical analysis

The data analyses were performed using STATISTICA for Windows version 10 (Tulsa, OK, USA). The mean and SD values were calculated for each variable. For each test the personal best trial was used as a final score. A Kolmogorov-Smirnov test was used to test for normality of the data. The average inter-item correlation coefficients (IIR) and Cronbach’s alpha reliability coefficients (CA) were used to determine the inter-subject reliability of the applied tests. The within-subject variation for each of the tests was determined by calculating the coefficient of variation (CV). To observe a systematic bias between individual trials (items) for each test, an ANOVA for repeated measures and a Tukey post-hoc test were used.

Linear correlation analyses were applied to detect the intercorrelations between the tests. Factor analysis (principal component analysis) was applied to define the latent structure of the used tests. The statistical significance was set at p*<0.05*.

## Results

By means of the Kolmogorov-Smirnov test, all variables were identified as normally distributed. The between subject-reliability was appropriate as indicated by high values of Cronbach’s alpha coefficients (from 0.91 to 0.97), and inter-item correlation coefficients (from 0.78 to 0.92). The within-subject variations (CVs) in the speed tests ranged between 1.3% and 9.1%, with the lowest variability (i.e. highest reliability) for the S30 test. In general, the lowest reliability was evidenced for the RCRAB (6.5%, 0.91, and 0.78; for CV, CA and IIR, respectively). The highest reliability was found in BS30 tests (2.8 %; 0.97 and 0.92; for CV, CA and IIR, respectively). Significant systematic biases between the trials were found in all of the tests except BEARC. In general, ANOVA revealed improvement from the 1st to 3rd testing trial, which indicates familiarization between testing trials. However, the post-hoc analyses reached statistical significance only between the first trial and the other two trials (for FCRAB, RCRAB, LT, and BS30) and between the first and second trials in S30. There were no significant differences between the 2nd and 3rd trial in any of the applied tests. Therefore, regardless of the ANOVA significance, we can highlight the stabilization of the results until the last (i.e. the third) testing trial. As a result, the final result for each examinee was expressed as the best achievement for each test (Table 1).

The intercorrelations between the variables ranged from low and non-significant values (0.28) to the moderate ones (r=0.71). Of all applied tests the S30 was numerically mostly related to other studied variables (Sum of correlations is 2.75). Meanwhile, the lowest inter-correlations were evidenced for BEARC (Sum of correlations is 1.70). The intercorrelations between the quadrupedal performances were more inconsistent and generally lower (from r=0.28 up to r=0.56) in comparison to the inter-correlations between bipedal performances (from r=0.53 up to 0.71). The factor analysis extracted only one significant factor with the highest projections of the S30 and BS30 (Table 2).

## Discussion

The 30 m sprinting from a static start timed by electronic timing gates is often used for assessing running speed, and the sprinting results of our study are within the range of previously reported data (Young et al., 2008; Chaouachi et al., 2009; Green et al., 2011). More precisely, our subjects achieved results better by 7% than non-elite rugby union players, and worse by 2–3% than elite rugby union players, professional basketball players and elite Australian Rules footballers (Green et al., 2011; Chaouachi et al., 2009; Young et al., 2008). However, previous studies have not reported data for other test performances that were used in the present study and therefore, those results cannot be compared.

Studies conducted so far reported high values of the inter-subject-reliability and within-subjects-reliability-coefficients of the sprint measures over distances up to 30 m (Chaouachi et al., 2009; Meylan et al., 2009; Green et al., 2011). Therefore, the high values of reliability parameters of 30-m sprint test in our study are in concordance with previous findings.

To the best of our knowledge, this is the first investigation that systematically studied the reliability of “non-forward” running speed tests. Evident differences can be observed between the CV values of quadrupedal and bipedal locomotions. Lower CV values for bipedal performances (1.3 to 3.2) compared to quadrupedal performances (6.6 to 9.1) are mostly explainable by familiarity of the tests. In contrast to quadrupedalism, bipedalism is a regular locomotion of adult humans. Familiarity with the movement patterns is one of the crucial factors for achieving the high reliability of the test procedures (Sealey et al., 2010). Therefore, it is logical that the subjects were more familiarized to more common activities (i.e., bipedal), which consequently led to a lower CV (i.e., higher reliability) for bipedal tests.

If only observing the factor analysis results, and significance of the correlation, one could conclude that S30 can be used as a universal tool for testing linear speed of different forms of locomotion. However, S30 shares only 17 to 34% common variances (the values r² converted into percentages) with other performances in this study, with the exception of 50% of the common variance shared with BS30. In general, a larger percent of non-shared variance (> 50%) indicates that two studied variables possess specific or at least relatively independent qualities (Huck, 2008). In other words, apart from S30 and BS30, all applied tests should be observed as relatively independent performances. Therefore, our research clearly justifies not only the construction of the specific linear speed tests, but also indicates the necessity of systematic specific training of different types of linear speed. Nevertheless, a relatively high percentage of unexplained variance (approximately 50%) between the S30 and BS30 tests indicates that even these two performances (forward and backward sprinting), do not necessarily have to be observed as indices of a unique linear-speed-quality. Although previous studies (Devita and Stribling, 1991; Flynn and Soutas-Little, 1995) have failed to show a relationship between forward and backward running speeds, their findings of differences between these virtually similar locomotions (with regard to ranges of motion, intrinsic support lengths and stance times, the dominant type of muscle contraction, stride frequencies etc.) can provide insight into the obtained (empirical) specificity of S30 and BS30.

The inconsistent relationship between the tests of the same motor ability is not rare and has been previously reported for different qualities (Sporis et al., 2010; Sekulic et al., 2012; Brouwer et al., 1998; Meylan et al., 2009). For the purpose of our investigation the most interesting is the fact that the study which examined agility among elite junior male soccer players reported poor relationships between different agility performances (Sporis et al., 2010). In this study the authors reported low (−0.06) to moderate (0.55) correlations between 6 agility measures, demonstrating the real nature of the relationship between sprint and lateral (shuffle) speed. The fact that we have found a poor relationship between lateral-speed-performance (LT) and forward-sprint-running (S30) is clearly supportive to those findings. Therefore, it seems reasonable to conclude that differences in the locomotion forms (forward running, shuffling, zig-zag, etc.), may be observed as a factor that actually influences the relationships of applied agility measures.

Abitbol (1988) discussed quadrupedal-locomotion (crawling) as more energy-demanding than bipedal-locomotions, but to the best of our knowledge, there has been no study that addresses the issue of differences between various quadrupedal performances in human adults (e.g., a comparison of some biomechanical features). The low correlations between quadrupedal tests (correlations ranged from 0.28 to 0.56) showed that different quadupedal tests should be observed as highly specific, regardless of their similar physiological and anatomical backgrounds. The explanation of low relationships between quadrupedal tests is probably multifactorial. First, it is clear that bipedal locomotions are relatively natural, practiced throughout everyday life and sport activities, and therefore – well learned. Oppositely, quadrupedal locomotion forms are rarely used, and therefore relatively complicated. Also, the quadrupedal movements that we have investigated require diverse involvement of the multi-axial shoulder and wrist joints, and thus, are kinematically more complex than bipedal locomotions.

## Conclusions

Based on the results of this study the following conclusions can be drawn. First, our results showed that all of the applied tests were reliable, relatively stable and therefore applicable in testing of subjects similar to those sampled herein. Second, it appears that different forms of linear (locomotion) speed should not be observed as unique quality, since inter-correlations between the tests varied considerably. This clearly reinforces the construction and validation of activity-specific tests. More precisely, one must be aware of the fact that the specificity of linear speed locomotion is not only determined by anatomical-circumstances (i.e., involvement of muscle groups, joints, etc.), but also by specificity of movement patterns for each tested quality (i.e., forward, backward, lateral, etc.). Finally, it is likely that inconsistency of the relationships between bipedal-movement-patterns explains the inconsistencies of the relationships between different agility tasks, which is noted but not sufficiently explained in the literature. The findings of this study should be applied in training programmes aimed at improving different types of agility performances.

## Figures and Tables

**Table 1:** Descriptive statistics and reliability coefficients for various tests of linear speed

Test	Mean±SD	CA	IIR	CV(%)	ANOVA (F)
FCRAB_trial1_ (s)	9.68±1.68	0.96	0.86	7.0	14.76^*^
FCRAB_trial2_ (s)	9.16±1.72^T1^				
FCRAB_trial3_ (s)	8.92±1.82^T1^				
FCRAB (s)^f^	8.65±1.52				
RCRAB_trial1_ (s)	7.41±1.10	0.91	0.78	6.5	14.02^*^
RCRAB_trial2_ (s)	7.00±0.91^T1^				
RCRAB_trial3_ (s)	6.89±0.89^T1^				
RCRAB (s)^f^	6.70±0.90				
BEARC_trial1_ (s)	9.82±1.40	0.97	0.94	9.1	1,28
BEARC_trial2_ (s)	9.77±1.51				
BEARC_trial3_ (s)	9.92±1.73				
BEARC (s)^f^	9.47±1.49				
LT_trial1_ (s)	4.19±0.26	0.92	0.81	3.2	21.14^*^
LT_trial2_ (s)	4.06±0.30^T1^				
LT_trial3_ (s)	4.01±0.31^T1^				
LT (s)^f^	3.96±0.30				
S30_trial1_ (s)	4.30±0.17	0.96	0.90	1.3	4.93^*^
S30_trial2_ (s)	4.26±0.18^T1^				
S30_trial3_ (s)	4.29±0.18				
S30 (s)^f^	4.24±0.17				
BS30_trial1_ (s)	6.20±0.61	0.97	0.92	2.8	10.19^*^
BS30_trial2_ (s)	6.10±0.56^T1^				
BS30_trial3_ (s)	6.04±0.50^T1^				
BS30 (s)^f^	5.97±0.51				

SD – standard deviation; CA – Cronbach Alpha; IIR – average inter item correlation coefficient; CV –coefficient of variation; FCRAB – linear speed test of supine forward (quadrupedal) locomotion; RCRAB- linear speed test with supine backward (quadrupedal) locomotion; BEARC- linear speed test of pronate backward (quadrupedal) locomotion; LT – lateral shuffle linear speed test; S30 – 30-m sprint test; BS30- backward 30-m sprint test;

f – fastest trial;

* - denotes significant differences at p < 0.05;

^T1^ – significantly different from trial1

**Table 2:** Intercorrelations between tests of linear speed and results of factor analysis

	FCRAB	RCRAB	BEARC	LT	S30	BS30		F
FCRAB	-	0.36^*^	0.56^*^	0.25	0.53^*^	0.33^*^		−0.69
RCRAB	0.36^*^	-		0.32^*^	0.41^*^	0.42^*^		−0.62
BEARC	0.56^*^	0.28	-	0.30	0.52^*^	0.32^*^		−0.67
LT	0.25	0.32^*^	0.30	-	0.58^*^	0.53^*^		−0.68
S30	0.53^*^	0.41^*^	0.52^*^	0.58^*^	-	0.71^*^		−0.88
BS30	0.33^*^	0.42^*^	0.32^*^	0.53^*^	0.71^*^	-		−0.77
								
Sum of R	2.03	1.79	1.70	1.98	2.75	2.31		
								
Expl Var								3.18
Prp Totl								0.53

* denotes significant Pearson’s correlation coefficients (p<0,05); FCRAB –linear speed test of supine forward (quadrupedal) locomotion; RCRAB- linear speed test with supine backward (quadrupedal) locomotion; BEARC-linear speed test of pronate backward (quadrupedal) locomotion; LT – lateral shuffle linear speed test, S30 – 30-m sprint test; BS30-backward 30-m sprint test; Sum of R – sum of intercorrelations for each test; F – factor structure; Expl Var – factor’s variance; Prp Totl – percentage of the explained variance
